# Adult *Ascaris suum* roundworms and their antigens modulate the porcine intestinal microbiome and metabolome

**DOI:** 10.1016/j.crpvbd.2026.100441

**Published:** 2026-09-22

**Authors:** Andrea Springer, Liane Wagner, Sarina Koehler, Stefanie Klinger, Michael Wendt, Gerhard Breves, Dagmar A. Brüggemann, Christina Strube

**Affiliations:** a Institute for Parasitology, Centre for Infection Medicine, University of Veterinary Medicine Hannover, Buenteweg 17, Hanover, 30559, Germany; b Max Rubner-Institut, Federal Research Institute of Nutrition and Food, Department of Safety and Quality of Meat, E.-C.-Baumann-Straße 20, Kulmbach, 95326, Germany; c Institute for Physiology and Cell Biology, University of Veterinary Medicine Hannover, Bischofsholer Damm 15, Hanover, 30173, Germany; d Clinic for Swine, Small Ruminants, Forensic Medicine and Ambulatory Service, University of Veterinary Medicine Hannover, Bischofsholer Damm 15, Hanover, 30173, Germany

**Keywords:** Ascarids, Ascariosis, Ascariasis, Soil-transmitted helminths, Intestinal microbiota, Excretory-secretory antigens, Short chain fatty acids

## Abstract

The porcine roundworm, *Ascaris suum*, affects host intestinal physiology, microbiota composition and metabolic profiles. To better understand the mediators of these alterations, six pigs each were experimentally exposed to (i) 30 adult roundworms *via* gastric tube, or (ii) received injections into the duodenum of *A. suum* total excretory-secretory (ES) antigens, ES antigens secreted exclusively from the parasites’ body surface (trans-cuticular ES), or cuticular somatic antigens, respectively. Six further pigs served as untreated controls. Microbiome composition in faeces, ingesta and mucosa samples from different intestinal compartments three days after transfer was studied by next-generation 16S rDNA sequencing. Metabolite profiles were assessed in ingesta and serum samples by nuclear magnetic resonance. Transfer of adult worms and their antigens significantly affected the caecal and colonic microbiome. Adult worm transfer also led to significantly increased levels of short-chain fatty acids (SCFAs) in the colon. Similarities between pigs exposed to adult *A. suum* and their antigens included increased relative abundance of operational taxonomic units (OTUs) representing potentially beneficial bacteria like *Lactobacillus* spp., *Prevotella* spp., and *Eubacterium coprostanoligenes*, while some opportunistic pathogens like *Citrobacter* spp. and *Mucispirillum schaedleri* were also increased. The net health effect during natural *A. suum* infections requires further study, but these preliminary findings warrant the hypothesis that low *A. suum* burdens might promote intestinal health. The results further indicate that certain microbial alterations are indeed mediated by *A. suum* antigens, although SCFA levels were unaffected in the antigen-treated groups, possibly due to the single application. Further investigations on chronically exposed animals are warranted, especially as porcine *A. suum* infections serve as a model for human ascariosis.

## Highlights

• Transfer of adult *Ascaris suum* to naïve pigs affected the intestinal microbiota.
• Similar microbial alterations were induced by exposure to different adult *A. suum* antigens.
• Infected pigs may benefit from increased *Lactobacillus* spp. and *Prevotella* spp.
• Opportunistic pathogens, such as *Citrobacter* spp. and *Mucispirillum schaedleri*, may also profit from infection.
• Adult worm transfer led to increased levels of short-chain fatty acids in the colon.

## Graphical abstract

**Figure:**
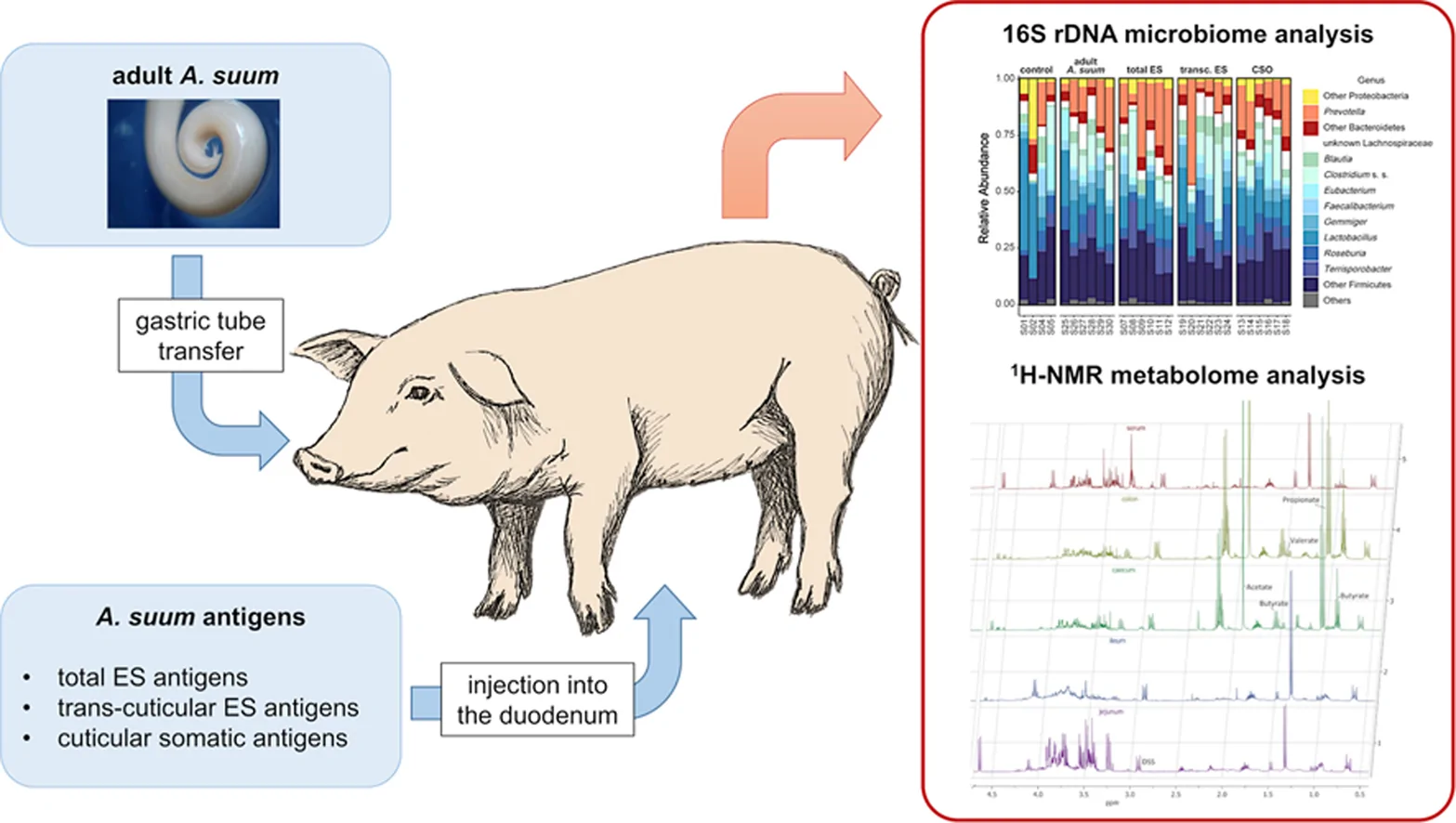

## 1 Introduction

Malnutrition and impaired development are hallmarks of gastrointestinal roundworm infection (O’Lorcain and Holland, 2000; Stephenson et al., 2000). The soil-transmitted helminth *Ascaris lumbricoides* affects approximately 800 million people worldwide (Global Burden of Disease Study 2013 Collaborators, 2015) and is recognized as a major cause of nutritional deficiencies, especially in children (O’Lorcain and Holland, 2000). Likewise, infections with the porcine parasite *Ascaris suum*, closely related to or even conspecific with *A. lumbricoides* (Leles et al., 2012; Zhou et al., 2020), may result in reduced feed conversion and weight gain in pigs (Thamsborg et al., 2013).

*Ascaris* spp. infections have a profound effect on the host’s intestinal physiology. After hatching of ingested eggs, the larvae penetrate the intestinal mucosa and undergo a body migration *via* the liver and lungs, before returning to the intestinal tract. Further development to adult stages then takes place in the lumen of the jejunum (Roepstorff et al., 1997). Although most immature stages are eliminated *via* a local immunological reaction approximately three weeks after infection (Masure et al., 2013), longevity of remaining worms can reach more than one year (Olsen et al., 1958). Consequences of infection include induction of a predominantly T-helper cell type 2-driven immune response, changes in intestinal gene expression and nutrient uptake (Dawson et al., 2005; Solano-Aguilar et al., 2018; Koehler et al., 2021a), effects on the intestinal microbiota (Williams et al., 2017; Wang et al., 2019; Springer et al., 2022), and metabolic changes (Wagner et al., 2026). Specifically, *A. suum* affects intestinal microbial diversity, stability of the microbiota over time and the abundance of short-chain fatty acid (SCFA)-producing taxa (Williams et al., 2017; Wang et al., 2019; Springer et al., 2022) and their metabolites in the large intestine (Wagner et al., 2026). Further, metabolic changes in the serum, including reduced glucose levels, indicate a profound effect on host energy metabolism (Wagner et al., 2026).

It seems likely that effects of *A. suum* on the microbiota are mediated by the parasites’ excretory-secretory (ES) products, as these show antibacterial properties and inhibit biofilm formation *in vitro* (Midha et al., 2018). It has been suggested that the worms thereby create a favourable metabolic environment and indirectly manipulate the intestinal immune system (Midha et al., 2021). However, the diversity of released antimicrobial factors varies between different *A. suum* life stages (Midha et al., 2018). The above-mentioned microbial and metabolic alterations have been observed during the first seven weeks of the infection (Williams et al., 2017; Wang et al., 2019; Springer et al., 2022; Wagner et al., 2026), corresponding roughly to the duration of prepatent infection, which lasts approximately six to eight weeks (Nansen and Roepstorff, 1999). Similar experimental studies on chronic infections with adult *Ascaris* spp. are not available so far. Therefore, it remains to be determined whether the presence of adult worms alone induces intestinal dysbiosis, or whether the microbial perturbation is a transient feature accompanying worm establishment. A few studies have assessed microbiota alterations in patently infected humans (Cooper et al., 2013; Lee et al., 2014; Rosa et al., 2018); however, the generated insights are limited due to potentially confounding effects of co-infections, continuous reinfections and exclusive reliance on faecal samples.

To gain more insight into the potential drivers of *Ascaris*-induced dysbiosis and the metabolic consequences, the present study exposed helminth-free pigs to adult *A. suum* by oral gastric tube application, thereby circumventing the larval body migration and larval development. Furthermore, different *A. suum* antigen fractions were injected into the duodenum of pigs to examine whether these substances *per se* induce microbial and metabolic changes or whether the presence of live adult parasites is required.

## 2 Materials and methods

### 2.1 Treatment of pigs and sample collection

*Ascaris suum* total ES antigens were derived by *in vitro* culture of adult worms (male-female sex ratio 1:2) in RPMI medium as previously described (Koehler et al., 2021b). Transcuticular ES antigens were obtained from an equal number of worms whose mouth and anal openings had been sealed with surgical tissue glue. Worms were cultured without addition of antibiotics, and collected medium was sterile-filtered before pooling and volume reduction by lyophilisation (Koehler et al., 2021b). Cuticular somatic (CSO) antigens were obtained by homogenizing *A. suum* cuticles shock-frozen in liquid nitrogen and dissolving the resulting powder in double-distilled water (Koehler et al., 2021b).

Animal experiments have been previously described in detail by Koehler et al. (2023). Briefly, 30 helminth-free pigs of *c.*30 kg/10 weeks of age were divided into five groups (*n* = 6 per group). On study day 0, pigs from the first group each received 30 adult roundworms (male-female sex ratio 1:2) by oral transfer *via* gastric tube. To verify successful worm establishment, these pigs were housed individually after worm transfer, to allow individual collection of all faeces until necropsy. The faeces were rinsed through a sieve to collect any expelled *A. suum* specimens. Further, faecal samples from the day of slaughter (study Day 3) were copromicroscopically examined for the presence of *A. suum* eggs.

Three further groups were subjected to transfer of total ES antigens, transcuticular ES antigens and CSO antigens, respectively, by injection into the duodenum during a laparotomic procedure. Thereby, each pig received the amount of total ES/transcuticular ES antigens produced by 30 worms over three days, or the amount of CSO antigens corresponding to 30 *A. suum* cuticles (male-female sex ratio 1:2). Pigs in the fifth group were left untreated and served as controls.

Faecal samples were taken rectally three days prior to treatment (study Day −3) and upon slaughter (study Day 3). Blood was sampled during exsanguination and centrifuged at 2000× *g* for 10 min to obtain serum. However, from three pigs no serum was available. Intestinal content was taken at necropsy with sterile tools from the proximal jejunum (*c.*3 m distal to the pylorus), medial jejunum (*c.*6–8 m distal to the pylorus), ileum, caecum and the second coil of the colon ascendens, and frozen immediately in liquid nitrogen. For one pig in the adult *A. suum* group, no proximal jejunum content was available. Intestinal mucosa was sampled at the same locations by rinsing a ∼5 × 3 cm piece of intestinal tissue with sterile 0.9% NaCl solution and stripping the mucosa with a glass slide, before snap-freezing in liquid nitrogen. All samples were stored at −80 °C until further processing.

### 2.2 Microbiome analysis

#### 2.2.1 DNA isolation, 16S rDNA amplification and sequence processing

DNA isolation was performed with the DNeasy® PowerLyzer® PowerSoil® kit (Qiagen, Hilden, Germany) for faecal samples and the QIAamp® BiOstic® Bacteremia DNA Kit (Qiagen, Hilden, Germany) for ingesta samples as described by Springer et al. (2022). For mucosa samples, the DNeasy® Blood and Tissue Kit (Qiagen, Hilden, Germany) was used according to the manufacturer’s instructions, including enzymatic pre-treatment for Gram-positive bacteria. The concentration and quality of extracted DNA were assessed using a NanoDrop™ 1000 instrument (PEQLAB Biotechnologie, Erlangen, Germany). Next-generation sequencing, sequence processing, operational taxonomic unit (OTU) clustering and taxonomy assignment were performed by a commercial laboratory (Microsynth AG, Balgach, Switzerland) as previously described by Springer et al. (2022). Briefly, samples were standardized to a DNA concentration of 2.5 ng/μl. Primers 341F and 805R were used to create two-step Nextera PCR libraries of the V3-V4 region of the bacterial 16S rRNA gene (Herlemann et al., 2011), which were sequenced on an Illumina MiSeq platform (Illumina Inc., San Diego, CA, USA). After de-multiplexing and adaptor trimming (MiSeq Reporter software v.2.6), read quality was checked with FastQC v.0.11.8 (Babraham Institute, Cambridge, UK), and primers were trimmed with cutadapt v.2.8 (Martin, 2011). The reads were further trimmed to 250 bp from the 3′-end to reduce sequencing noise, before merging with USEARCH v.11.0.667 (Edgar, 2010). Merged sequences were quality filtered allowing for a maximum of one expected error per merged read, discarding reads with ambiguous bases and outliers regarding amplicon size. Denoising was performed *via* the UNOISE algorithm (Edgar and Flyvbjerg, 2015) and the resulting OTU table was filtered for possible bleed-in contaminations using UNCROSS (Edgar, 2016b). Taxonomies were predicted with the RDP 16S reference database (Cole et al., 2014) using the SINTAX algorithm implemented in USEARCH (Edgar, 2016a). OTU clustering and taxonomic assignment were performed separately for faecal samples, mucosa samples as well as for small intestine (jejunum, ileum) and large intestine (colon, caecum) ingesta samples, resulting in four separate datasets.

#### 2.2.2 Statistical analysis of microbiome data

The microbiome dataset was analysed in R v.4.0.2 (R Core Team, 2021), using the packages *phyloseq* (McMurdie and Holmes, 2013), *vegan* (Oksanen et al., 2019), *DESeq2* (Love et al., 2014) and *lmerTest* (Kuznetsova et al., 2017). After inspection of rarefaction curves, the faecal and ingesta datasets were rarefied to 8000 reads/sample, and the mucosa dataset to 1500 reads/sample, i.e. samples with less sequences were removed from further analyses. Alpha diversity, i.e. the microbial diversity within each sample, was assessed by calculating the number of observed OTUs as well as Chao1, Shannon and inverse Simpson indices, whereby the latter two take not only the absence/presence, but also the abundance of OTUs into account (Morris et al., 2014). Alpha diversity of faecal samples before and after experimental treatment was compared within each group by Wilcoxon signed rank tests for paired data. Alpha diversity values of ingesta samples and mucosa samples were compared between experimental groups for each intestinal segment using the Kruskal-Wallis test with FDR-correction of *P*-values, followed by Dunn’s *post-hoc* test as appropriate.

To assess differences in microbiota composition (i.e. beta diversity), permutational multivariate analysis of dispersions (PERMDISP, a multivariate analogue of Levene’s test for homogeneity of variance) and permutational analysis of variance (PERMANOVA, 10,000 permutations) were carried out based on Jensen-Shannon distances (JSD). For faecal samples, the PERMANOVA included the experimental group, time point (before or after treatment) and their interaction as predictors, and was stratified by animal ID to account for repeated sampling. For ingesta and mucosa samples, separate PERMANOVA were run only for ileum and large intestine datasets, due to low sequencing success of jejunum samples, whereby the large intestine models contained the predictor “compartment” (caecum/colon) in addition to “experimental group” as well as their interaction, and the stratification term “animal ID”.

The relative abundance of microbial phyla as well as genera was compared between faecal samples taken before and after treatment in each group using Wilcoxon signed rank tests for paired data with FDR-correction of *P*-values. For ingesta and mucosa samples, Kruskal-Wallis tests were conducted, followed by Dunn’s *post-hoc* tests if corrected *P*-values were ≤ 0.05. Furthermore, differences in OTU-level abundance were investigated by *DESeq2* differential expression analyses, based on unrarefied datasets as *DESeq2* performs an internal normalization (Love et al., 2014).

### 2.3 Metabolome analysis

#### 2.3.1 Sample preparation of ingesta and serum

Homorganic ingesta samples (100 mg; proximal and medial jejunum, ileum, caecum, colon) were thawed at room temperature for 30 min, moved to a polystyrene tray with ice, mixed with 800 μl MilliQ water and vortexed two times for 10 s at 4 m/s using a bead mill homogenizer (Bead Ruptor Elite, OMNI International, NW Kennesaw, USA). The samples were then centrifuged at 14,000× *g* (Hettich centrifuge, Mikro 200R, Hettich GmbH, Tuttlingen, Germany) for 5 min at 4 °C. A volume of 350 μl of filtrate was transferred to a standard 5 mm glass NMR tube (Wilmad, LabGlass, USA) with 150 μl of D_2_O (deuterium oxide, 99.9%, Cambridge Isotope Laboratories, Andover, MA, USA) and 50 μl of phosphate buffer (pH = 7.4) containing 10 mM DSS (3-(trimethylsilyl)-1-propanesulfonic acid-d_6_ sodium salt (Sigma-Aldrich, St. Louis, MO, USA) as internal standard for quantification and chemical shift reference.

Serum samples (300 μl) were thawed at room temperature for 30 min, moved to a polystyrene tray with ice, vortexed, and filtered through prewashed Nanosep centrifugal filters with 3 kDa cut-off (Pall Life Science, Port Washington, NY, USA) by centrifugation at 12,000× *g* for 90 min at 4 °C. Then, 200 μl filtrate was mixed with 300 μl phosphate buffer (pH = 7.4) containing 10 mM DSS and 50 μl D_2_O and transferred to an NMR tube.

#### 2.3.2 ^1^H-NMR spectroscopy and data processing

All samples (ingesta and serum) were analysed with a 400 MHz NMR spectrometer (Bruker BioSpin GmbH, Rheinstetten, Germany) operating at a proton NMR frequency of 400.31 MHz for ^1^H and equipped with a 5 mm TXI probe. Tuning and shimming were performed for each sample.

The 1D NOESY pulse experiment with presaturation of the spectral region containing the water peak (noesygppr1d) was used with a recycle delay of 6.5 s. A total of 32 scans were acquired, and the acquisition parameters included 65,536 data points, a spectral width of 8223.69 Hz, and an acquisition time of 3.98 s. Measurements were done at 300 K. ^1^H-^1^H correlation spectroscopy (COSY), ^1^H-^1^H total correlation spectroscopy (TOCSY) and ^1^H-^13^C heteronuclear single quantum correlation (HSQC) were obtained on one representative sample for metabolite identification purposes.

All data were processed using Bruker Topspin v.4.0.7 software (Bruker BioSpin GmbH, Rheinstetten, Germany). An experimental window function of 0.3 Hz was applied to all datasets before Fourier transformation. After the NMR spectra were manually phase- and baseline-corrected, each NMR spectrum was further analysed using MATLAB (v.R2018, MathWorks Inc., Natic, MA, USA). In MATLAB, data were normalized to the area of DSS and referenced to standard peak DSS at 0.00 ppm. Regions that contained no metabolite signals (above 9 ppm and below 0.5 ppm) and the interval containing the residual water signal (5.00–4.70 ppm) were excluded. Finally, the spectra were binned into 0.01 ppm intervals (buckets). Afterwards, the NMR signals were identified using ChenomX NMR Suite 8.4 library (ChenomX Inc., Edmonton, AB, Canada), the Human Metabolome Database (www.hmdb.ca) and previous literature (Bankefors et al., 2011; Wagner et al., 2014) and confirmed with 2D-NMR in case of multiplicity.

#### 2.3.3 Multivariate and univariate statistical analyses

For multivariate data analyses, the preprocessed ^1^H NMR spectral data were imported into SIMCA-P+ software (v.18.2; Sartorius Stedim Data Analytics AB, Umetrics AB, Umeå, Sweden), centered and “pareto”-scaled. Principal components analysis (PCA) was used to gain an overview of the data and search for outliers using PCA-Hotelling T^2^ Ellipse (95% confidence interval (CI)) and DModX (95% CI). Hotelling’s T^2^ identifies observations with unusually high leverage within the PCA model, whereas DModX measures the distance of an observation from the model in the residual space. Samples exceeding both the respective 95% CI thresholds were considered outliers and excluded from further analysis. The multivariate data were checked by normal probability plot of the PCA model for normal distribution. Orthogonal partial least squares projection to latent structures-discriminant analysis (OPLS-DA), a supervised technique, was performed for classification of different treatments. OPLS-DA loading plot and variable influences in projection (VIP) plot for spectral regions with VIP > 1 and with jack-knife-based CI that did not include unity were considered to be discriminative between the different groups. Cross validation (CV) analysis of variance (ANOVA) (*P* ≤ 0.05) for significance testing of OPLS-DA model was used (Eriksson et al., 2008). The signals that contributed most to the differences between the groups (acetate, butyrate, propionate and valerate in the colon) were quantified, and their concentrations (μmol/mg) were calculated using the ChenomX NMR Suite v.8.4 profiler. The absolute concentrations of the four metabolites were tested for differences between the five groups using the statistical software JMP (v.17.0.0; SAS Institute Inc., Cary, NC, USA). For univariate statistical testing of absolute metabolite concentrations, data normality was assessed using the Shapiro-Wilk test. Due to non-normal distribution in metabolites, parametric ANOVA was not applied. Instead, the non-parametric Kruskal-Wallis test was used to compare the five groups. When this test showed a significant effect (*P* ≤ 0.05), *post-hoc* pairwise comparison between the five groups (control *vs* adult *A. suum*; control *vs* total ES; control *vs* transcuticular ES; control *vs* CSO) was conducted by multiple Mann-Whitney tests. All data are presented as the mean ± standard deviation (SD) and differences were considered significant when *P* ≤ 0.05.

Correlations between SCFA (acetate, butyrate, propionate and valerate) levels and relative abundance of microbial genera in the colonic ingesta were assessed using Spearman’s rank correlation with FDR-correction of *P*-values.

## 3 Results

### 3.1 Parasitological analyses

On the day of necropsy, the six pigs infected by transfer of 30 adult *A. suum* were copromicroscopically positive for *A. suum* eggs. Examination of their faeces revealed expulsion of three worms one day prior to necropsy in one pig, and two worms each in two pigs (Koehler et al., 2023). All control animals and the antigen-exposed animals remained copromicroscopically negative.

### 3.2 Microbiome analysis

DNA concentration prior to standardization ranged from 6.3 to 250.6 ng/μl (median: 150.3 ng/μl), from 2.1 to 2798.8 ng/μl (median: 203.6 ng/μl), and from 54.1 to 1260.8 ng/μl (median: 287.4 ng/μl) for faecal, ingesta and mucosa sample extracts, respectively. Sequencing success differed among sample types. Of the initial 34,250,851 merged sequences generated during this study, 6.3% (2,146,343) were discarded during the quality control workflow. While only one faecal sample (a pre-treatment sample from the total ES antigen group) was excluded after quality filtering, only 53 of 89 available small intestinal ingesta samples (9/29 from the proximal jejunum, 15/30 from the medial jejunum, 29/30 from the ileum) and 53 of 60 large intestinal ingesta samples (28/30 from the caecum, 25/30 from the colon) were successfully sequenced. Sequencing success was even lower for mucosa samples, as only 6 of 90 samples from the small intestine (1/30 from the proximal jejunum, 1/30 from the medial jejunum and 4/30 from the ileum) and 46 of 60 samples from the large intestine (24/30 from the caecum, 22/30 from the colon) yielded a sufficient amount of bacterial sequences. Therefore, only ingesta samples from the ileum, caecum and colon, and mucosa samples from the caecum and colon were analysed further. An overview of samples included in the final analysis from each experimental group is given in Table 1.

**Table 1:** Overview of samples per experimental group included in the final analysis of microbiome and metabolome alterations in pigs after transfer of adult *Ascaris suum* worms or *A. suum* total excretory-secretory (ES), transcuticular ES, or cuticular somatic (CSO) antigens.

	Control	Adult *A. suum*	Total ES	Transcuticular ES	CSO
**Microbiome analysis**					
Faecal samples (study day −3)	6	6	5	6	6
Faecal samples (study day 3)	6	6	6	6	6
Ingesta ileum	5	6	6	6	6
Ingesta caecum	4	6	6	6	6
Ingesta colon	4	6	5	4	4
Mucosa caecum	4	6	3	6	5
Mucosa colon	4	5	4	5	4
**Metabolome analysis**					
Ingesta proximal jejunum	6	5	6	6	6
Ingesta medial jejunum	6	6	6	6	6
Ingesta ileum	6	6	6	6	6
Ingesta caecum	6	6	6	6	6
Ingesta colon	6	6	6	6	6
Serum	6 (3)^a^	6 (4)^a^	5	6 (5)^a^	4

a Outliers found in PCA plot, number of samples after their exclusion from further analyses.

#### 3.2.1 Microbiome analysis of faecal samples

The faecal sample dataset contained 2,581,026 sequences (mean ± SD sequences per sample: 43,746 ± 22,541). After rarefying to 8000 reads per sample, all 59 samples were retained, containing 455 unique OTUs (11 phyla, 33 families, 73 genera). The dominating phyla in pig faeces were Firmicutes and Bacteroidetes, with a mean relative abundance of 68.1% (SD: 9.4%) and 25.5% (SD: 6.8%) per sample, respectively.

When comparing faecal samples taken before and after exposure to adult *A. suum* or their antigens, no statistically significant differences in alpha diversity were detected (Table 2). Regarding beta diversity, PERMDISP indicated no significant differences in dispersion before and after treatment within each experimental group. PERMANOVA analysis only revealed a significant effect of time point, but no differences between experimental groups and no significant interaction effect (Table 3, Fig. 1A).

**Table 2:** Summary of microbial alpha diversity metrics of porcine faecal samples taken three days before and three days after transfer of adult *Ascaris suum* worms or *A. suum* total excretory-secretory (ES), transcuticular ES, or cuticular somatic (CSO) antigens.

	Control	Adult *A. suum*	Total ES	Transcuticular ES	CSO
**Observed OTUs**					
Pre-treatment (mean ± SD)	295.67 ± 24.64	280.17 ± 84.49	229.40 ± 63.87	274.83 ± 43.44	285.67 ± 22.62
Post- treatment (mean ± SD)	288.00 ± 28.83	291.17 ± 38.70	311.00 ± 25.19	290.33 ± 37.81	292.50 ± 13.23
Wilcoxon test: V, *P*-value	18.00, 0.156	11.00, 1.000	1.00, 0.125	6.00, 0.787	8.50, 0.753
**Chao1 index**					
Pre-treatment (mean ± SD)	308.05 ± 24.61	291.29 ± 91.41	235.60 ± 67.17	286.76 ± 41.39	296.36 ± 30.38
Post- treatment (mean ± SD)	295.73 ± 29.69	305.68 ± 48.51	325.35 ± 29.68	297.13 ± 40.17	308.61 ± 16.29
Wilcoxon test: V, *P*-value	19.00, 0.094	11.00, 1.000	1.00, 0.125	9.00, 0.844	6.00, 0.438
**Shannon index**					
Pre-treatment (mean ± SD)	4.33 ± 0.12	4.29 ± 0.42	4.17 ± 0.53	4.49 ± 0.57	4.55 ± 0.23
Post- treatment (mean ± SD)	4.51 ± 0.24	4.44 ± 0.19	4.46 ± 0.24	4.55 ± 0.22	4.36 ± 0.26
Wilcoxon test: V, *P*-value	6.00, 0.438	7.00, 0.563	4.00, 0.438	11.00, 1.000	17.00, 0.219
**Inverse Simpson index**					
Pre-treatment (mean ± SD)	22.17 ± 5.35	26.67 ± 8.37	31.29 ± 23.19	47.87 ± 33.74	41.91 ± 16.8
Post- treatment (mean ± SD)	34.03 ± 17.62	31.71 ± 10.96	31.50 ± 14.13	36.72 ± 17.68	30.82 ± 14.35
Wilcoxon test: V, *P*-value	3.00, 0.156	7.00, 0.563	6.00, 0.813	13.00, 0.688	16.00, 0.313

*Abbreviation*: SD, standard deviation.

**Table 3:** Results of PERMDISP and PERMANOVA analyses on the microbiota composition of porcine faecal samples taken three days before and three days after transfer of adult *Ascaris suum* worms or *A. suum* total excretory-secretory (ES), transcuticular ES, or cuticular somatic (CSO) antigens. Significant *P*-values (*P* ≤ 0.05) are printed in bold.

	*df*	SS	MS	*F*	*R*^2^	*P*
**PERMDISP (control)**						
Day (post- *vs* pre-treatment)	1	0.002	0.002	0.941	–	0.355
Residuals	10	0.022	0.002	–	–	–
**PERMDISP (adult *A. suum*)**						
Day (post- *vs* pre-treatment)	1	0.000	0.000	0.003	–	0.957
Residuals	10	0.040	0.004	–	–	–
**PERMDISP (total ES)**						
Day (post- *vs* pre-treatment)	1	0.012	0.012	1.401	–	0.267
Residuals	9	0.075	0.008	–	–	–
**PERMDISP (transcuticular ES)**						
Day (post- *vs* pre-treatment)	1	0.011	0.011	1.687	–	0.223
Residuals	10	0.064	0.006	–	–	–
**PERMDISP (CSO)**						
Day (post- *vs* pre-treatment)	1	0.007	0.007	1.317	–	0.278
Residuals	10	0.055	0.005	–	–	–
**PERMANOVA**						
Group	4	0.479	0.120	2.865	0.168	0.089
Day (post- *vs* pre-treatment)	1	0.151	0.151	3.615	0.053	**0.009**
Group*day interaction	4	0.174	0.043	1.040	0.061	0.459
Residuals	49	2.048	0.042	–	0.718	–
Total	58	2.852	–	–	1.000	–

*Note*: As PERMDISP is a univariable test, this was calculated for each group separately, while for PERMANOVA the experimental groups could be combined in a single test.

*Abbreviations*: *df*, degrees of freedom; SS, sum of squares; MS, mean squares.

**Fig. 1:**
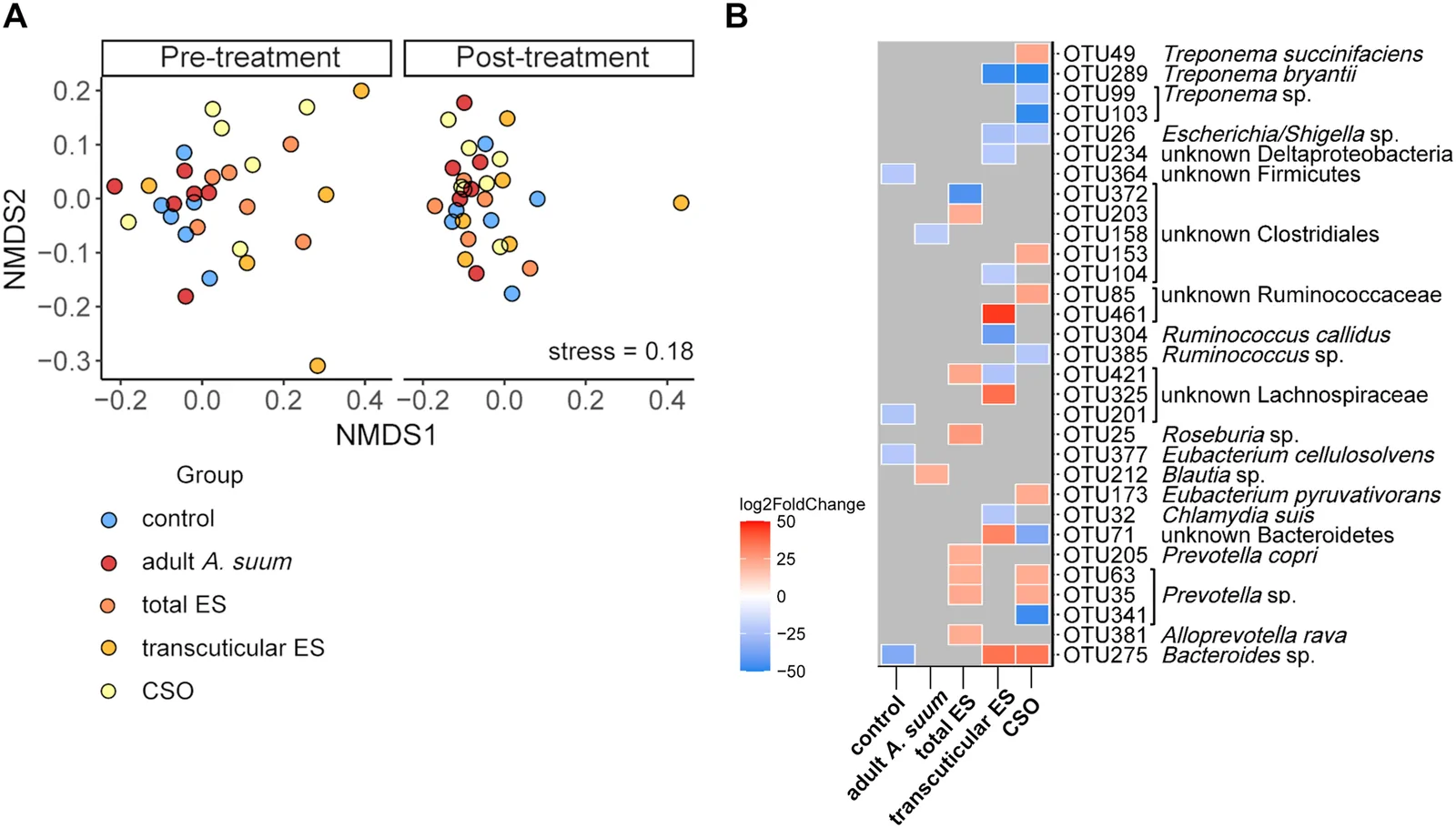
Changes in the faecal microbiota of pigs three days after transfer of adult *Ascaris suum* worms or their total excretory-secretory (ES), transcuticular ES, and cuticular somatic (CSO) antigens *vs* an untreated control group. **A** Non-metric multidimensional scaling (NMDS) plot of microbial community composition in pre- and post-treatment faecal samples. The stress value of 0.18 indicates an acceptable 2D representation of the original high-dimensional data. **B** Heatmap of differentially abundant OTUs in post-*vs* pre-treatment faecal samples as determined by DESeq2 analysis. Only differences with Benjamini-Hochberg adjusted *P*-values ≤ 0.01 and a minimum log_2_ fold change of ± 20 are shown as coloured tiles.

No significant differences in relative abundance were detected at the phylum, family and genus level when comparing pre- and post-treatment faecal samples (Wilcoxon signed rank test for paired data, FDR-corrected *P*-values > 0.05). At the OTU level, only few significant changes with a minimum log_2_ fold change of ± 20 were determined by DeSeq2 analysis, with little consistencies between groups (Fig. 1B).

#### 3.2.2 Microbiome analysis of ingesta and mucosa samples

Ingesta samples yielded a total of 13,862,994 (mean 261,566 ± 170,326 per sample) sequences for the small intestine, and 15,190,334 (mean 286,610 ± 176,434) sequences for the large intestine. Upon rarefication to 8000 reads/sample, two colon samples were excluded. The rarefied datasets contained 107 unique OTUs (4 phyla, 16 families, 33 genera) from the small intestine and 814 unique OTUs (9 phyla, 36 families, 82 genera) from the large intestine. The 46 mucosa samples from the large intestine contained 447,720 sequences (mean 9733 ± 7034), and 219 OTUs (8 phyla, 31 families, 61 genera) after rarefication to 1500 reads/sample.

The ingesta of the ileum was dominated by the phylum Firmicutes, with mean relative abundance of 90.8% (SD: 13.8%). In the caecum, Firmicutes and Bacteroidetes showed a mean relative abundance of 77.3% (SD: 11.8%) and 18.3% (SD: 11.4%), similar to the colon with 80.3% (SD: 8.8%) and 17.1% (SD: 8.8%). The mucosal microbiota of the caecum and colon mainly comprised Firmicutes (mean relative abundance of 57.0%, SD: 20.81%) and Proteobacteria (mean 27.1%, SD: 18.6%).

#### 3.2.3 Microbial alpha and beta diversity: Ileum

A significant difference in alpha diversity was noted in the ileum with regard to the number of observed OTUs and the Chao1 index (Kruskal-Wallis test, *χ*^2^ = 12.61 and 14.20, respectively, *df* = 4, FDR-adjusted *P* = 0.027 in both cases), which were significantly higher in pigs exposed to CSO compared to the control group (Dunn’s *post-hoc* test, *P* = 0.006 and 0.002, respectively; Fig. 2A). In terms of beta diversity, PERMDISP and PERMANOVA analyses indicated no significant differences in overall microbiota composition between the different experimental groups in ileal ingesta (Table 4).

**Fig. 2:**
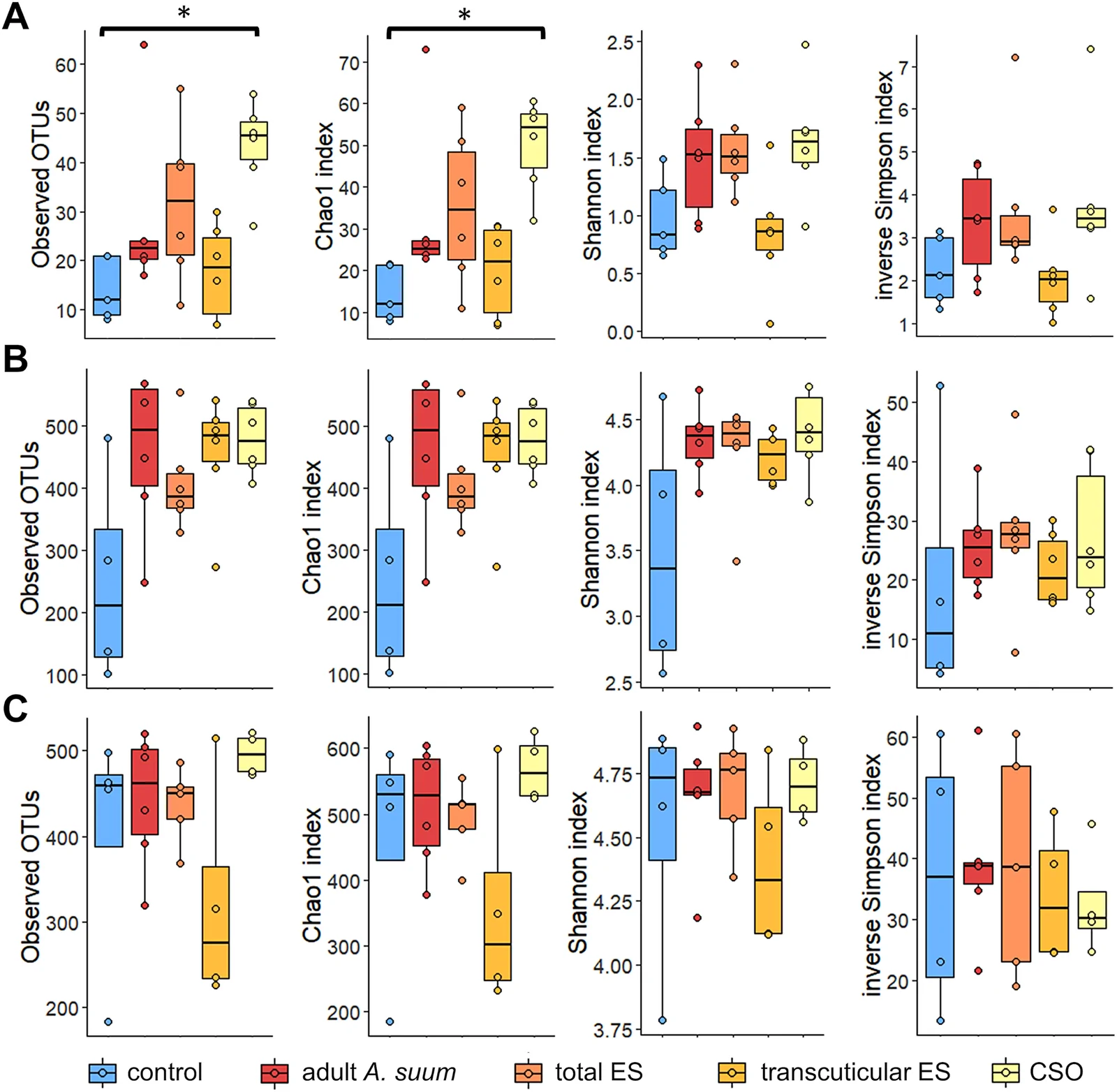
Microbial alpha diversity in porcine ingesta samples from the ileum (**A**), caecum (**B**) and colon (**C**) three days after exposure to adult *Ascaris suum* worms or their total excretory-secretory (ES), transcuticular ES, and cuticular somatic (CSO) antigens *vs* an untreated control group. Individual data points as well as boxplots are shown, with boxes extending from the 25th to the 75th percentile, a line at the median and whiskers extending to 1.5 the interquartile range. Statistically significant differences are indicated by asterisks.

**Table 4:** Results of PERMDISP and PERMANOVA analyses on the microbiota composition of porcine ingesta and mucosa samples taken three days after transfer of adult *Ascaris suum* worms or *A. suum* total excretory-secretory (ES), transcuticular ES, or cuticular somatic (CSO) antigens, compared to a control group. Significant *P*-values (*P≤*0.05) are printed in bold.

	Ingesta						Mucosa (large intestine only)					
	*df*	SS	MS	*F*	*R*^2^	*P*	*df*	SS	MS	*F*	*R*^2^	*P*
**PERMDISP (ileum)**												
Group	4	0.06	0.01	0.51	–	0.733	–	–	–	–	–	–
Residuals	24	0.66	0.03	–	–	–	–	–	–	–	–	–
**PERMANOVA (ileum)**												
Group	4	0.23	0.06	0.83	0.12	0.559	–	–	–	–	–	–
Residuals	24	1.69	0.07	–	0.88	–	–	–	–	–	–	–
Total	28	1.93	–	–	1.00	–	–	–	–	–	–	–
**PERMDISP (caecum)**^a^												
Group	4	0.07	0.02	1.65	-	0.206	4	0.12	0.03	3.74	–	**0.021**
Residuals	18	0.19	0.01	–	–	–	19	0.15	0.01	–	–	–
**PERMDISP (colon)**^a^												
Group	4	0.11	0.03	2.35	-	0.084	4	0.02	0.01	0.69	–	0.6088
Residuals	23	0.27	0.01	–	–	–	17	0.13	0.01	–	–	–
**PERMANOVA (caecum + colon)**												
Compartment (caecum vs. colon)	1	0.17	0.17	3.06	0.06	**<0.001**	1	0.43	0.43	6.46	0.12	**<0.001**
Group	4	0.47	0.12	2.13	0.16	**<0.001**	4	0.79	0.20	2.97	0.21	**0.038**
Compartment*group interaction	4	0.05	0.01	0.24	0.02	0.979	4	0.10	0.03	0.39	0.03	0.880
Residuals	41	2.25	0.05	–	0.77	–	36	2.40	0.07	–	0.64	–
Total	50	2.94	–	–	1.00	–	45	3.73	–	–	1.00	–

*Abbreviations*: *df*, degrees of freedom; SS, sum of squares; MS, mean squares.

a As PERMDISP is a univariable test, this was calculated for caecum and colon separately, while for PERMANOVA both compartments could be combined in a single test.

#### 3.2.4 Microbial alpha and beta diversity: Caecum and colon

Microbial alpha diversity tended to be higher in the caecal ingesta of the exposed groups compared to the control group, although the differences were not statistically significant (Fig. 2B). Regarding mucosa samples of the large intestine, no significant differences in alpha diversity were detected between the experimental groups (Kruskal-Wallis test, FDR-adjusted *P* > 0.05).

For the large intestine, PERMDISP indicated a significant difference in dispersion only with regard to the mucosa-associated microbiota in the caecum, whereas PERMANOVAs indicated significant differences according to the compartment as well as the experimental group in both ingesta and mucosa samples. Interestingly, the effect of experimental group was larger than the effect of compartment (*R*^2^ of 0.16 *vs* 0.06 for ingesta, and 0.21 *vs* 0.12 for mucosa samples; Table 4, Fig. 3). When performing PERMANOVAs for ingesta samples of each experimental group *vs* the control group separately, a significant difference was detected for the adult *A. suum* and the total ES antigens group, but not for the transcuticular ES and CSO groups (Supplementary Table S1). Regarding mucosa samples, all but the total ES antigen-exposed group showed a significant difference to the control group (Supplementary Table S1).

**Fig. 3:**
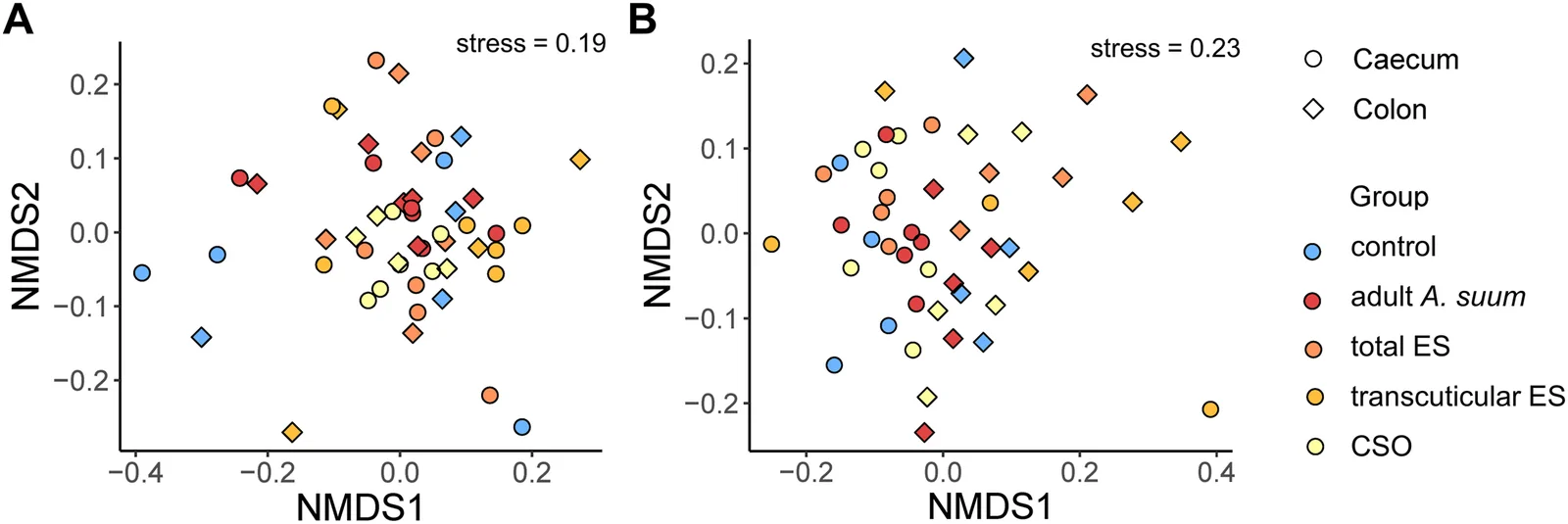
Non-metric multidimensional scaling (NMDS) plot of microbial community composition in large intestinal ingesta (**A**) and mucosa (**B**) samples of pigs exposed to adult *Ascaris suum* worm or their total excretory-secretory (ES), transcuticular ES, and cuticular somatic (CSO) antigens *vs* an untreated control group. Intestinal compartment is indicated by different shapes, while colour denotes the experimental group. The stress value of 0.19 in panel **A** indicates an acceptable 2D representation of the original high-dimensional data, while the higher stress value of 0.23 in panel **B** indicates that the distances between points should be interpreted with caution.

#### 3.2.5 Changes in microbial relative abundance

No significant differences in relative abundance at the phylum, family or genus level were detected between the experimental groups in ingesta and mucosa samples of any intestinal compartment (Kruskal-Wallis tests, FDR-corrected *P*-values > 0.05; Fig. 4). At the OTU level, an overall pattern of increased relative abundance was noted in the ileal and caecal ingesta of all *A. suum* worm- and antigen-exposed pigs as compared to the control group. Particularly, in all exposed groups, OTUs assigned to *Streptococcus hyointestinalis* were increased in both the ileum and caecum, and OTUs assigned to different *Lactobacillus* spp., except *Lactobacillus pontis*, and *Citrobacter* spp. were increased in the ileum (Supplementary Fig. S1). In the caecum, OTUs assigned to *Prevotella* spp. were mostly increased across all exposed groups (Supplementary Fig. S1). In the colon, a more mixed pattern of increased and decreased abundance was observed, with a consistent increase of OTUs belonging to *Eubacterium coprostanoligenes* and a consistent decrease of OTUs assigned to *Clostridium XIVa* spp. in all exposed groups. Further, the abundance of one OTU assigned to *Anaerovibrio* spp. increased in all exposed groups in both caecum and colon (Supplementary Fig. S1).

**Fig. 4:**
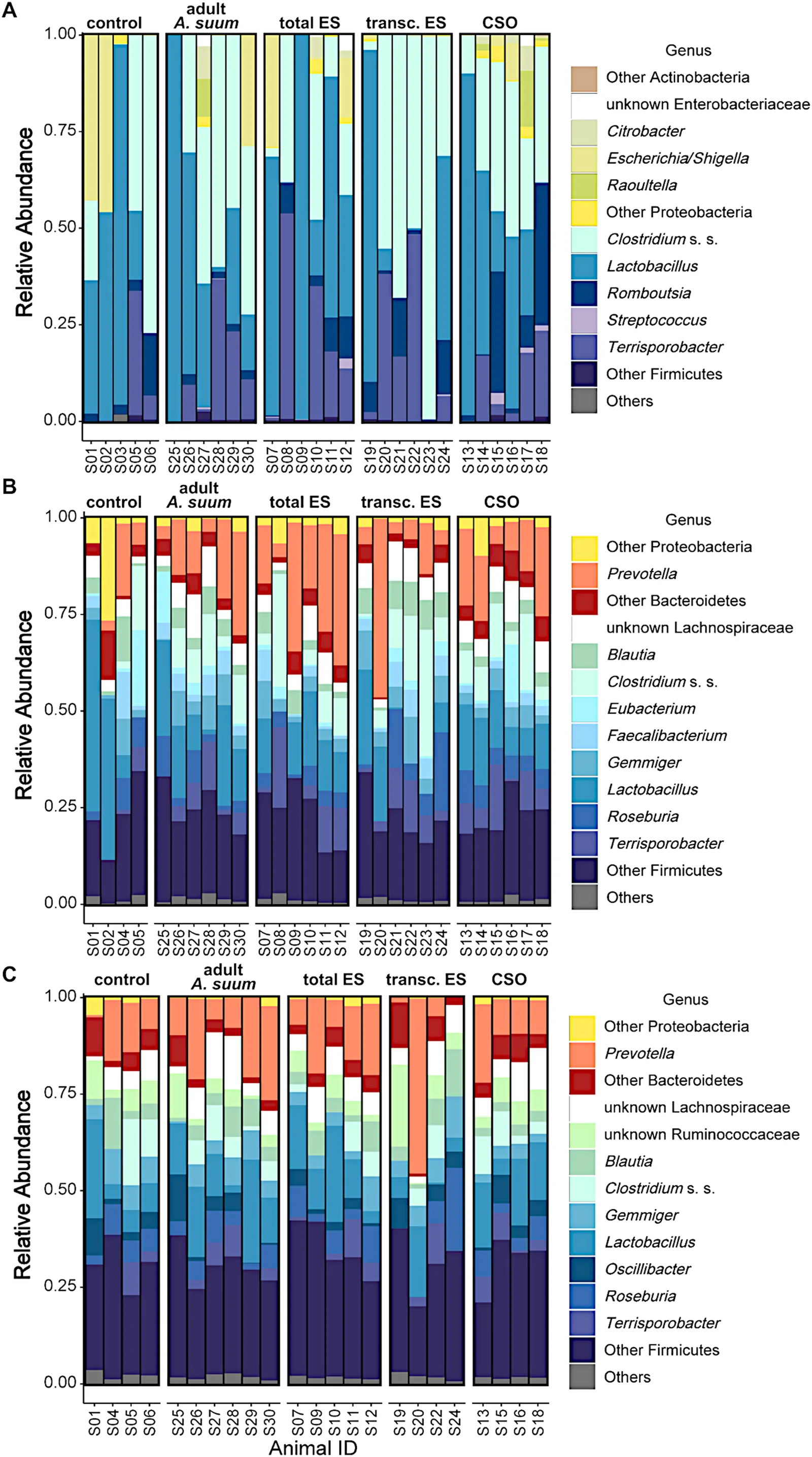
Genus-level microbiota composition in porcine ingesta of the ileum (**A**), caecum (**B**), and colon (**C**) three days after exposure to adult *Ascaris suum* worms or their total excretory-secretory (ES), transcuticular ES, and cuticular somatic (CSO) antigens *vs* an untreated control group.

In contrast to the ingesta samples, only a few significant changes in OTU abundance were noted regarding the mucosa-associated microbiome of the caecum and colon, but an enrichment of *Mucispirillum schaedleri* in the colon was a consistent finding, except after treatment with transcuticular ES antigens (Supplementary Fig. S1).

### 3.3 ^1^H NMR-based metabolomic analysis

Representative ^1^H NMR spectra from jejunum, ileum, caecum and colon extracts of a pig exposed to *A. suum* CSO are shown in Fig. 5. To examine variations in metabolic profiles of pigs exposed to adult roundworms and their different antigen fractions compared to untreated control samples, as well as between ingesta from different intestinal compartments and serum, NMR spectral data were analysed using PCA (PC1 *vs* PC2). The dataset included 149 ingesta samples and 27 serum samples (Table 1), as one proximal jejunum sample from the adult *A. suum* group and one serum sample from the total ES antigens group, as well as two serum samples from the CSO group, were not available. All PCA and OPLS-DA model parameters, including number of components, R2X, Q2, R2Y, and CV-ANOVA, are provided in Supplementary Table S2.

**Fig. 5:**
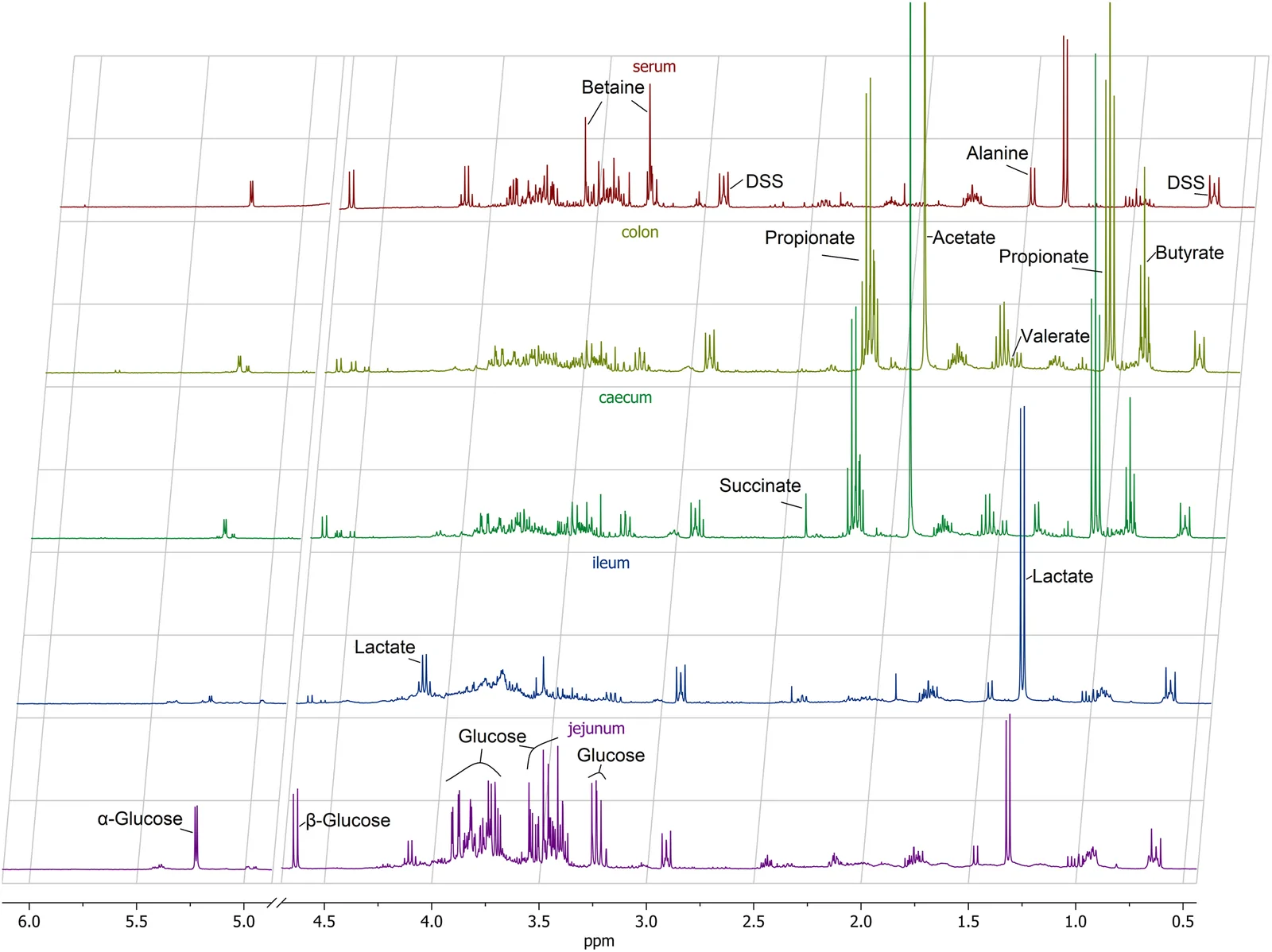
Representative ^1^H NMR spectra (0.5–6.0 ppm) of ingesta extracts from jejunum, ileum, caecum, colon and serum extracts of a pig exposed to *A. suum* cuticular somatic antigen (CSO).

#### 3.3.1 Metabolic changes in the ingesta

In the PCA model (Fig. 6), the first and second components explained 66.4% and 12.0% of the variation, respectively, and the total amount of variation was 98.3% using 18 principal components. A clear separation between the different intestinal compartments in terms of jejunum, ileum, and large intestine was observed (Fig. 6A). An OPLS-DA model, which is a supervised method to confirm the results obtained by PCA, showed no significant difference between the experimental groups. Further exploration within the PCA model, focusing on the different treatments, independent of intestinal compartment, showed no clear separation (Fig. 6B). Therefore, the data analyses were continued individually for each compartment.

**Fig. 6:**
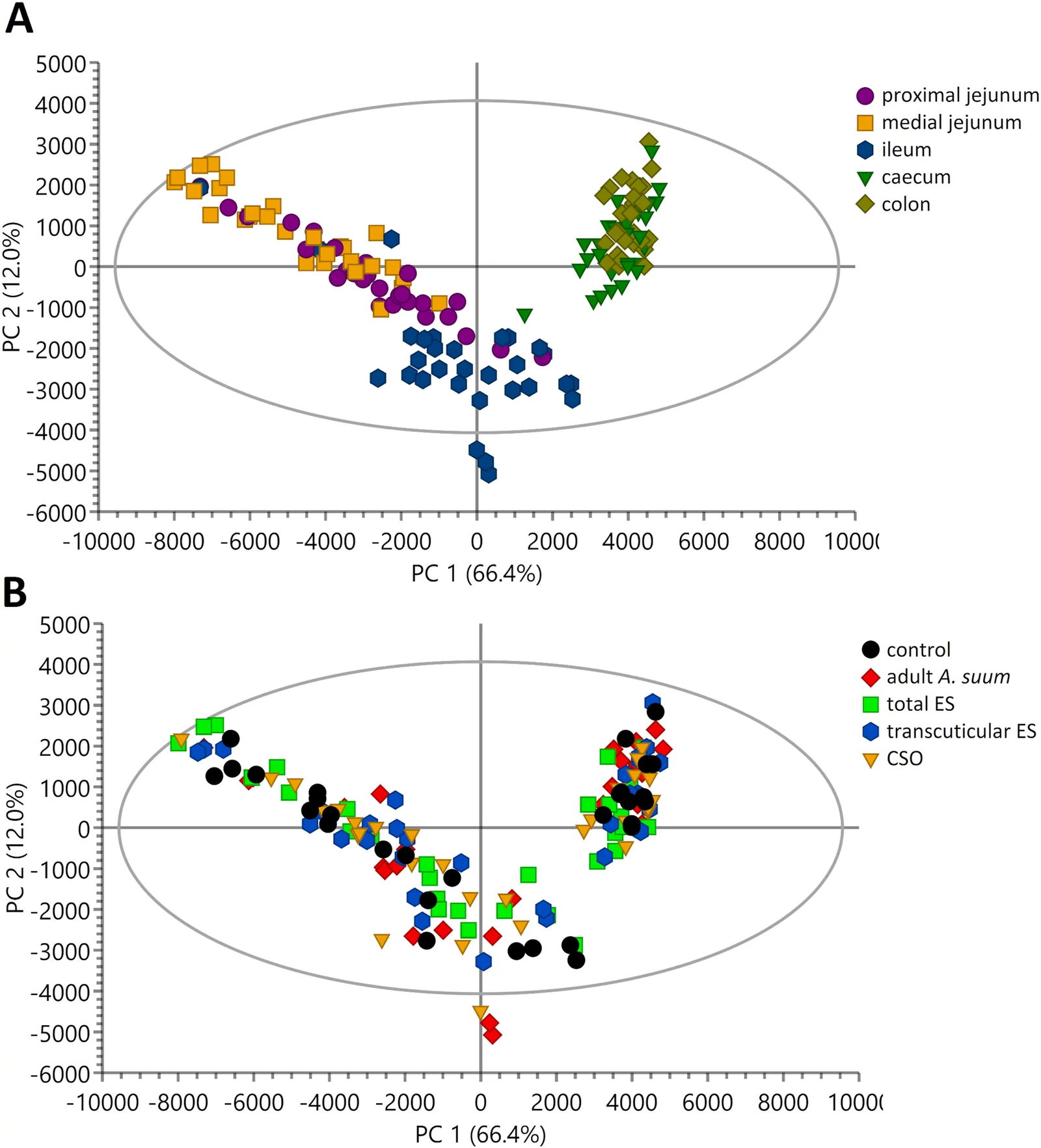
The principal components analysis (PCA) score plots from ^1^H NMR spectrum profiles of ingesta samples from pigs exposed to adult *A. suum* worms or their total excretory-secretory (ES), transcuticular ES, and cuticular somatic (CSO) antigens *vs* an untreated control group, coloured according to intestinal compartment (**A**) and experimental group (**B**).

##### 3.3.1.1 Ingesta - jejunum

The PCA model for proximal and medial jejunum was described by 18 PCs, of which the first and second components described 61.4% and 13.8% of variation, respectively, and showed no clear separation (Fig. 7A). Although an OPLS-DA model could be generated, it did not provide additional information. Therefore, pairwise comparisons (treatment groups *vs* untreated control group) were performed. The score plots of the PCA models showed no separation between the different exposed pigs and the control group. Also, the OPLS model showed no significant differences.

**Fig. 7:**
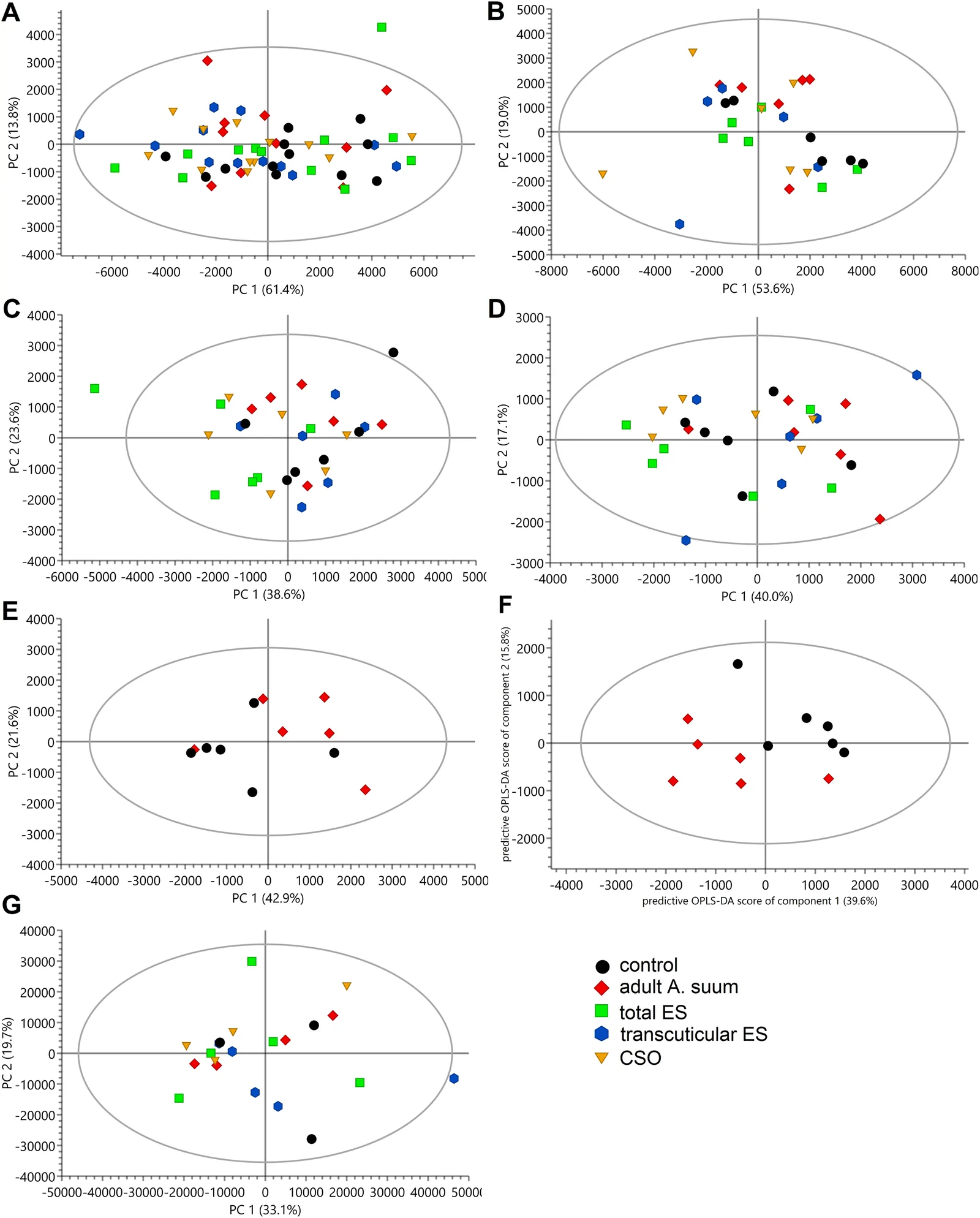
The principal components analysis (PCA) score plots from ^1^H NMR spectrum profiles of ingesta samples from the jejunum (**A**), ileum (**B**), caecum (**C**), colon (**D**), and serum (**G**) samples, and of pigs exposed to adult *A. suum* worms or their total excretory-secretory (ES), transcuticular ES, and cuticular somatic (CSO) antigens *vs* an untreated control group. A PCA score plot (**E**) and OPLS-DA score plot (**F**) of colon samples from pigs exposed to adult *A. suum* worms *vs* the untreated control group are additionally shown.

##### 3.3.1.2 Ingesta - ileum

The PCA model for the ileum resulted in eight components, explaining a total amount of 96.9% of variation. The first component explained 53.6% of spectral variation and the second 19.0% in the PCA model (Fig. 7B). The OPLS-DA model did not provide further insights, similar to the results obtained for the jejunum. Likewise, pairwise comparisons between each treatment group and the control group did not uncover additional insights.

##### 3.3.1.3 Ingesta - caecum

The PCA model for the caecal ingesta was explained by two PC, where PC1 explained 38.6% and PC2 explained 23.6% of variation (Fig. 7C). The score plot of the OPLS-DA model using one component (1+0+0) showed no separation between the groups. Consistent with the findings for the jejunum and ileum, additional pairwise comparisons revealed no significant differences between the treatment groups and the untreated control group.

##### 3.3.1.4 Ingesta - colon

In the PCA model for the colon, the first and second PC explained 40.0% and 17.1% of variation, respectively, and the total amount of variation was 90.6% using seven principal components (Fig. 7D). It was not possible to generate an OPLS-DA model. Therefore, pairwise comparisons were conducted. The PCA and OPLS-DA models showed no separation between the treatment groups and the untreated control group, except for the adult *A. suum* group. In the PCA score plot of the adult *A. suum vs* untreated control group, the first of three PC described 42.9% of the variation, while the second PC explained 21.6% of spectral variation. The plot suggested a separation of both groups (Fig. 7E). The score plot of the generated OPLS-DA model using two predictive and one orthogonal component achieved a separation between the two groups (Fig. 7F). For determination of the NMR signals causing the differences, the VIP plot and loading column plot of PLS-DA were inspected. The spectral region with VIP > 1, for which the corresponding jack-knife-based confidence intervals were not close to or including zero, were considered to have high reliability and were chosen as discriminative. The NMR-based metabolomics analyses of colon extracts showed a significantly higher concentration of valerate, propionate, butyrate and acetate in pigs inoculated with adult *A. suum* worms compared with the untreated control group (Fig. 8, Table 5).

**Fig. 8:**
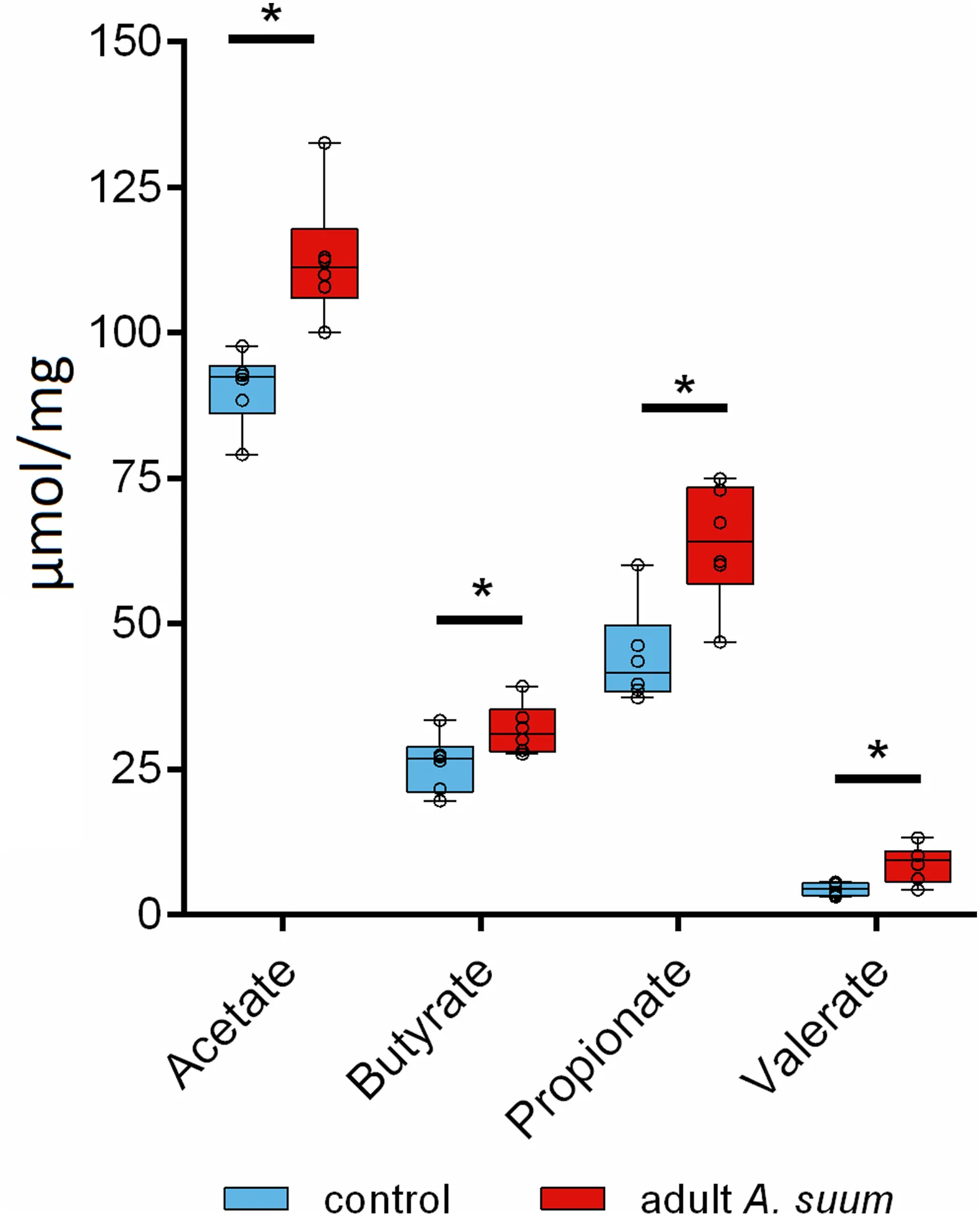
Concentrations of short-chain fatty acids in the colon content of pigs exposed to adult *A. suum* worms *vs* an untreated control group. Individual data points as well as boxplots are shown, with boxes extending from the 25th to the 75th percentile, a line at the median and whiskers extending to 1.5 the interquartile range. Statistically significant differences are indicated by asterisks.

**Table 5:** Short-chain fatty acid concentrations (μmol/mg) in the colon extract of pigs of the untreated control group and after transfer of adult *Ascaris suum* worms or *A. suum* total excretory-secretory (ES), transcuticular ES, or cuticular somatic (CSO) antigens. Significant *P*-values (*P≤*0.05) are printed in bold.

Fatty acid	Concentration in μmol/mg (Mean ± SD)					*P*
	Control	Adult *A. suum*	Total ES antigens	Transcuticular ES	CSO antigens	
Acetate	90.34 ± 5.81*	112.7 ± 10.84*	93.62 ± 14.93	98.60 ± 14.93	105.7 ± 17.59	**0.0034**
Butyrate	25.96 ± 4.46*	31.90 ± 4.31*	20.76 ± 9.14	30.04 ± 13.62	28.63 ± 6.19	**0.0184**
Propionate	45.62 ± 8.47*	63.87 ± 10.32*	48.96 ± 11.23	59.65 ± 11.58	49.11 ± 10.35	**0.0082**
Valerate	4.55 ± 1.03*	8.83 ± 3.19*	4.68 ± 3.29	7.32 ± 5.47	4.42 ± 2.10	**0.0184**

*Notes*: Mean values across rows marked with * are significantly different. *P*-values were calculated by Kruskal-Wallis/Mann-Whitney test.

*Abbreviation*: SD, standard deviation.

#### 3.3.2 Metabolic changes in serum

Six outliers were found that exceeded the Hotelling’s T^2^ statistic and DModX threshold in the serum samples (three from control group, one from the transcuticular ES group and two from adult *A. suum* group) and were therefore excluded from further analysis. The analysis of the remaining 21 serum samples resulted in a five-component model that explained 84.4% of the total amount of variation, where PC1 explained 33.1% and PC2 explained 19.7% of variation (Fig. 7G). It was not possible to create an OPLS-DA model, due to insufficient separation between the groups.

#### 3.3.3 Correlation between microbial genera and SCFAs

For the ingesta of the colon, the association between microbial genera and acetate, butyrate, propionate as well as valerate levels was assessed. The genus *Megasphaera* was significantly positively correlated with valerate levels (Spearman’s *ρ* = 0.76, FDR-corrected *P* = 0.010), while the genus *Campylobacter* was negatively associated with propionate concentration (Spearman’s *ρ* = −0.69, FDR-corrected *P* = 0.041).

## 4 Discussion

To gain more insight into the potential drivers of roundworm-induced dysbiosis and the metabolic consequences, the present study exposed helminth-free pigs to adult *A. suum* by oral gavage *via* a gastric tube, thereby circumventing larval migration and development, as well as to adult *A. suum* total ES, transcuticular ES, and cuticular somatic (CSO) antigens by injection into the duodenum.

As the present study was the first of its kind, several limitations need to be kept in mind. Since the application of *A. suum* antigens required a laparotomic procedure, this was only performed once to limit the impact on animal welfare while allowing initial insights. Due to the single administration, a rather short exposure period of only three days was chosen, whereas some alterations might take longer to develop. Repeated or continuous exposure over longer time spans would be more representative of natural infections and should be pursued in further follow-up studies. Furthermore, larval migration and development were deliberately circumvented in the present study to delineate the impact of adult worms and their antigens. It should thus be kept in mind that the model does not mimic natural infections. Furthermore, the limited 16S rDNA sequencing success, particularly regarding small intestinal samples, resulted in unanticipated sample loss and reduced group sizes, limiting the study’s power to detect significant differences. This sample loss precluded the analysis of microbial changes in the jejunum, the place of residence of the adult parasites. Limited sequencing yield due to low bacterial abundance in the jejunum has been reported before (De Rodas et al., 2018; Springer et al., 2022). Therefore, no comparison with the results by Andersen-Civil et al. (2022) was possible, who observed significant changes in the porcine jejunal microbiota at day 14 post *A. suum*-infection.

Despite these limitations, significant differences between the treatment groups and the control group were apparent in ingesta taken from the ileum, caecum and colon, whereas comparison of faecal samples taken three days before and three days after exposure to adult worms and their antigens revealed no significant changes in microbial diversity nor overall microbiota composition. In the ileum, microbial alpha diversity was significantly higher in the CSO-exposed group compared to the control group. In the caecum, alpha diversity tended to be higher in all exposed groups compared to the control group, although a statistically significant difference was not observed due to a high level of variation in the control group. In contrast, a transient reduction in microbial diversity occurred in the caecum of pigs single-infected with 10,000 *A. suum* eggs, but not in pigs subjected to trickle-infection over 10 days, at day 35 post (initial) infection (p.i.) (Springer et al., 2022). Wang et al. (2019) also observed a reduced microbial diversity in the colon of pigs at day 54 after infection with 300 *A. suum* eggs/day for three consecutive days. Although the comparability of the present model with natural chronic infections is limited, it is noteworthy that an increased diversity has been reported in faeces of humans chronically infected with soil-transmitted helminths, including roundworms (e.g. Rosa et al., 2018; Rubel et al., 2020). These mixed results may indicate that adult roundworms and their antigens exert a different effect on microbial diversity than larval or preadult stages, rather favouring than decreasing diversity. However, given the short exposure period and single administration of the antigens in the present study, this needs to be confirmed by further studies on chronically infected pigs.

Furthermore, beta diversity analyses of the caecal and colonic ingesta as well as the mucosa-associated microbiome revealed significant differences between exposed groups. In detail, the overall microbiota composition in ingesta differed significantly between the control group and pigs exposed to adult *A. suum* as well as to total ES antigens, while the mucosa-associated microbiome was significantly affected by all exposures, except for total ES antigens. Although no significant differences were observed at the phylum, family or genus level, interesting parallels between pigs exposed to adult *A. suum* and their antigens, as well as with previous investigations, were observed in terms of altered OTU abundance. These similarities indicate that certain microbial alterations are indeed mediated by *A. suum* (ES) antigens, as suggested by their antimicrobial properties shown *in vitro* (Midha et al., 2018).

An overall pattern of predominantly increased abundance of several OTUs assigned to *Lactobacillus* spp., except for a decrease in, e.g. *L. pontis*, was noted in all *A. suum* (antigens) exposed groups, particularly in the ileum. In general, *Lactobacillus* spp. are regarded as probiotic bacteria with anti-inflammatory potential (Turroni et al., 2014), and their increase might thus be beneficial for both the host and the parasite. A similar expansion of *Lactobacillus* spp. was evident in the jejunum and colon of pigs infected with *A. suum* eggs at day 14 and 54 p.i. (Wang et al., 2019; Andersen-Civil et al., 2022), as well as in *Trichuris suis*-infected pigs at four weeks p.i. (Stolzenbach et al., 2020). In contrast, another study on pigs infected with *A. suum* eggs, performed during the first seven weeks of infection (day 21, 35 and 49 p.i.), yielded a mixed pattern of increased *Lactobacillus* spp. in faecal samples, but a decrease in certain species, such as *Lactobacillus equicursoris*, in ingesta samples (Springer et al., 2022). Of note, OTUs assigned to *L. equicursoris* were increased in the ileum in the present study, but showed a decreased abundance in the caecum and colon of pigs exposed to adult *A. suum*, similar to the findings by Springer et al. (2022). Thus, alterations were not uniform across the intestinal tract, and their net impact on animal health remains to be determined.

Like *Lactobacillus*, the genus *Prevotella* is considered predominantly beneficial for intestinal health and represents a keystone taxon in the pig intestine, mediating growth performance and disease resilience (Amat et al., 2020). *Prevotella* spp. are associated with fibre degradation and fermentative production of SCFAs with anti-inflammatory properties (Koh et al., 2016). In the present study, a predominant significant increase of OTUs assigned to *Prevotella* was observed in the caecal ingesta of all exposed groups, as well as in faecal samples of the total ES antigen exposed group, while in the colon rather a mixed pattern of up- and downregulated *Prevotella* OTUs prevailed. Thus, as with regard to *Lactobacillus*, alterations were not consistent across the intestinal tract. Wang et al. (2019) observed an increase of *Prevotella* in the porcine colon on day 54 post *A. suum*-infection, while a transient decrease on day 35 p.i. was reported by Springer et al. (2022). Increases in *Prevotella* have also been observed in chronically *A. lumbricoides*-infected humans in Thailand and Colombia (Toro-Londono et al., 2019; Klomkliew et al., 2022).

Despite the inconsistencies across the intestinal tract, the increase of *Lactobacillus* spp. and *Prevotella* spp. induced by adult *A. suum* and their antigens, respectively, in some intestinal compartments during the present study could imply that a balanced state limiting harm to the host while favouring parasite persistence may develop during roundworm infection. The increase of OTUs assigned to *Eubacterium coprostanoligenes*, which has been associated with strengthening the intestinal mucus barrier by promoting mucin production (Bai et al., 2024), in the colonic ingesta of all exposed groups may also contribute to mitigating host damage. In humans, this bacterial species promotes ulcerative colitis remission (Zakerska-Banaszak et al., 2025) and is discussed as a potential probiotic that may lower serum cholesterol levels, favouring cardiovascular health (Mukherjee et al., 2020). Moreover, OTUs assigned to *Streptococcus hyointestinalis* showed increased abundance in the ileum and caecum of all exposed groups. Different strains of this species have been shown to produce antimicrobial proteins that may inhibit potential pathogens, such as *Clostridium perfringens*, *Listeria monocytogenes* and *Staphylococcus aureus* (O’Connor et al., 2015; Heo et al., 2018). Overall, the expansion of several taxa with beneficial properties suggests that low-level *A. suum* infections might confer a health advantage, again keeping in mind that the present model is not entirely representative of natural infections due to the lack of larval passage and development. Nevertheless, this could be an underlying reason for the fact that not all immature worm stages are expelled during the early phase of the infection (Masure et al., 2013).

On the other hand, some OTUs belonging to potential pathogens also showed an increased abundance. An OTU assigned to *Anaerovibrio* spp. was increased in the caecum and colon of all exposed groups. This genus has been associated with diarrhea in piglets and may be a novel opportunistic pathogen (Yang et al., 2019; Karasova et al., 2021). Furthermore, OTUs assigned to the genus *Citrobacter* were consistently increased in the ileum of all exposed groups. *Citrobacter* spp. are considered opportunistic pathogens and have been associated with gastrointestinal inflammation, e.g. during *Salmonella* infection of pigs (Argüello et al., 2018), and in human patients with gastroenteritis and irritable bowel syndrome (Ganji et al., 2016; Khorsand et al., 2022). Their increase might reflect an inflammatory state of the ileal mucosa, in line with the upregulation of IL-13 transcription observed in these pigs (Koehler et al., 2023). Similarly, expansion of *Mucispirillum schaedleri*, which primarily colonizes the mucus layer of the colon, has been linked to intestinal inflammation (Herp et al., 2021). An OTU assigned to this species was increased in the mucosa-associated colonic microbiome in all exposed pigs, except for those treated with transcuticular ES antigen, possibly related to increased mucus production triggered by *E. coprostanoligenes*.

Moreover, a reduction of OTUs assigned to *Ruminococcus* was observed in the caecum and colon of pigs inoculated with adult *A. suum*. Like *Prevotella* spp., ruminococci are involved in the digestion of complex polysaccharides. Members of the family Ruminococcaceae were equally reduced in the colon of *A. suum*-infected pigs at day 54 p.i (Wang et al., 2019). and in stool samples of *A. lumbricoides*-infected humans (Toro-Londono et al., 2019). In the present study, the decreased OTUs predominantly belonged to *Ruminococcus callidus*, which utilizes di- and trisaccharides (e.g. cellobiose, glucose, maltose, lactose) and predominantly produces succinate and acetate (La Reau and Suen, 2018). A significant reduction of this species in Crohn’s disease (Kang et al., 2010) and gastric bypass (Wijdeveld et al., 2023) patients suggests that this species may contribute to intestinal health, and its reduction may be a negative consequence of *A. suum* infection.

In terms of metabolic alterations, elevated SCFA (acetate, butyrate, propionate and valerate) concentrations were measured in the colon three days after transfer of adult *A. suum* worms. In contrast, no significant changes in metabolite concentrations were detected after injection of the various adult *A. suum* antigens, despite the described changes in microbiome composition. This may be due to the single application of antigens three days prior to slaughter, whereas adult worms delivered the antigens continuously.

The increase of SCFAs in the colon is in line with previous research on pigs infected with *A. suum* eggs, with a similar SCFA increase in the colon on day 21 p.i., followed by a transient decrease on day 35 p.i., and partial recovery on day 49 p.i. (Wagner et al., 2026). In another study, increased SCFA concentrations were also noted on day 54 p.i. (Zaiss et al., 2015). In contrast, on day 14 p.i. no changes in the levels of propionate and butyrate were yet detected in the colon by Williams et al. (2017). Like the study by Wagner et al. (2026), the present study highlights the colon as the primary site of metabolic adaptation to *A. suum* infection, as no significant metabolic changes were detected in the other intestinal compartments.

Short-chain fatty acids not only represent an energy source for the host but also downregulate inflammatory processes in the intestine by affecting cytokine patterns and recruitment of leukocytes, among other mechanisms (Koh et al., 2016), and may thus contribute to a state of parasite tolerance (Gause and Maizels, 2016). Adult *A. suum* themselves are known to produce SCFAs (Moyle and Baldwin, 1952). However, it seems unlikely that the increased concentrations were due to production by the parasites, as they were measured in the colon and not in the small intestine, where *A. suum* resides. Nevertheless, metabolic products of *A. suum*, in addition to their antigens, may contribute to shaping the intestinal microbial community (Midha et al., 2021). Here, the relative abundance of the genus *Megasphaera* was strongly positively correlated with valerate levels in the colon. The type-species of this genus, *Megasphaera elsdenii*, predominantly metabolizes lactate to SCFAs, whereby some strains produce mainly valerate (Yoshikawa et al., 2018). In other strains, increased valerate production has been noted when both lactate and glucose are available (Marounek et al., 1989). Therefore, increased availability of lactate due to expansion of *Lactobacillus* spp. may have led to increased valerate production by members of *Megasphaera* in the present study. Furthermore, the genus *Campylobacter* was significantly negatively associated with propionate levels. In previous studies, *Campylobacter* spp. correlated negatively with various volatile fatty acids (Lv et al., 2020; Fan et al., 2022), and a bacteriostatic effect of propionate on *Campylobacter jejuni* has been observed *in vitro* (Van Deun et al., 2008).

NMR analysis of serum samples did not reveal any significant differences between the experimental groups. Of the SCFAs increased in the colon of the adult *A. suum*-group, butyrate is predominantly used locally by colonocytes and propionate is metabolized in the liver (Koh et al., 2016), so increased levels in the peripheral bloodstream were not necessarily expected. Furthermore, the exposure period to adult worms of three days may have been too short to induce major alterations in host metabolism, which is also reflected by the fact that no functional alterations of intestinal nutrient transport were observed in these pigs in Ussing chamber experiments (Koehler et al., 2023). In contrast, exposure to *A. suum* total ES and CSO antigens resulted in upregulated amino acid transport across the duodenal and jejunal mucosa, while glucose transport was increased in the duodenum after total ES antigen exposure. These upregulations may have been a compensatory reaction after a transient downregulation (Koehler et al., 2023). However, the NMR measurements in the present study indicated that this did not translate to significantly increased serum concentrations. Similarly, in pigs single-infected with 10,000 *A. suum* eggs, no significant serum metabolic alterations were seen at 21, 35, and 49 days p.i., whereas pigs trickle-infected with *A. suum* eggs showed serum metabolic changes consistent with changes observed in the colon (Wagner et al., 2026).

## 5 Conclusions

The present study showed that adult *A. suum* modulate the intestinal microbiome and metabolome within three days after being transferred into parasite-naïve pigs, and that certain microbial changes can be replicated by injecting the parasites’ antigens. Expansion of *Lactobacillus* spp. in the ileum, *Prevotella* spp. in the caecum, and *Eubacterium coprostanoligenes* as well as increased SCFA concentrations in the colon support the suggestion of a predominantly anti-inflammatory milieu induced by the presence of adult worms. Increased levels of *Streptococcus hyointestinalis* may protect against pathogenic bacteria, although some potential pathogens like *Citrobacter* spp. and *M. schaedleri* also seem to profit. However, the relevance of these findings for natural infections remains to be elucidated, particularly given the absence of larval migration and development and the short duration of the present study. Should the increase in beneficial bacteria prevail over potential pathogens also during natural infections, low *A. suum* worm burdens, as replicated in the present study, might confer a health advantage. This may be controversially discussed as an underlying reason for the fact that not all worms are expelled during the immunological “self-cure” reaction in the early phase of the infection. The colon appears as the primary place of metabolic adaptation to *A. suum* infection, as no significant metabolic changes were detected in the other intestinal compartments. Again, the fact that no systemic metabolic response was detected in the serum may be related to the short exposure period of only three days. Therefore, further studies on naturally and chronically exposed animals are necessary to confirm and expand the present findings, particularly as porcine *A. suum* infections serve as a model for human ascariosis.

## Ethical approval

Animal experiments were conducted in accordance with the EU Directive 2010/63/EU on the protection of animals used for scientific purposes and the German Animal Welfare Act, as well as national guidelines for animal welfare. The study was approved by the ethics commission of the Animal Care and Use Committee of the Lower Saxony State Office for Consumer Protection and Food Safety (*Niedersächsisches Landesamt für Verbraucherschutz und Lebensmittelsicherheit*) under reference number 33.19.42502-04-16/2272.

## Credit authorship contribution statement

**Andrea Springer:** Conceptualization, Investigation, Data curation, Formal analysis, Visualization, Writing - original draft. **Liane Wagner:** Investigation, Data curation, Formal analysis, Visualization, Writing - original draft. **Sarina Koehler:** Investigation, Writing - review & editing. **Stefanie Klinger:** Investigation, Writing - review & editing. **Michael Wendt:** Investigation, Writing - review & editing. **Gerhard Breves:** Investigation, Writing - review & editing. **Dagmar Brüggemann:** Conceptualization, Supervision, Writing - review & editing. **Christina Strube:** Conceptualization, Project administration, Funding acquisition, Supervision, Writing - review & editing.

## Funding

This work was financially supported by the 10.13039/501100001659Deutsche Forschungsgemeinschaft (German Research Foundation; Grant Number STR 1171/16-1).

## Declaration of competing interests

The authors declare the following financial interests/personal relationships that may be considered potential competing interests: Christina Strube reports financial support from Deutsche Forschungsgemeinschaft. The other authors declare that they have no known competing financial interests or personal relationships that could have appeared to influence the work reported in this paper.

## Data Availability

Sequence data of the 16S rRNA amplicon sequencing have been deposited in the National Centre for Biotechnology Information Sequence Read Archive (SRA) under the SRA accession: PRJNA1348442. The metabolomics data have been deposited in the MetaboLights (Yurekten et al., 2023) repository with the study identifier MTBLS14497.
