# Supplementary material for: Adult Ascaris suum roundworms and their antigens modulate the porcine intestinal microbiome and metabolome
Source: Curr Res Parasitol Vector Borne Dis. 2026 Sep 22;10:100441. doi: 10.1016/j.crpvbd.2026.100441 (PMC13634906; doi:10.1016/j.crpvbd.2026.100441)
Supplement: Supplementary file 1 [file mmc1.pdf]

Supplementary Figure S1. Heatmaps of differentially abundant OTUs in (**A**) ileal ingesta, (**B**) caecal ingesta, (**C**) colonic ingesta, (**D**) ceacal mucosa, and (**E**) colonic mucosa samples of pigs exposed to adult *A. suum* worms or their total excretory-secretory (ES), transcuticular ES, and cuticular somatic (CSO) antigens *vs* an untreated control group, as determined by DESeq2 analysis. Only differences with Benjamini-Hochberg adjusted *P*-values ≤ 0.01 and a minimum log2 fold change of ± 20 are shown as coloured tiles.

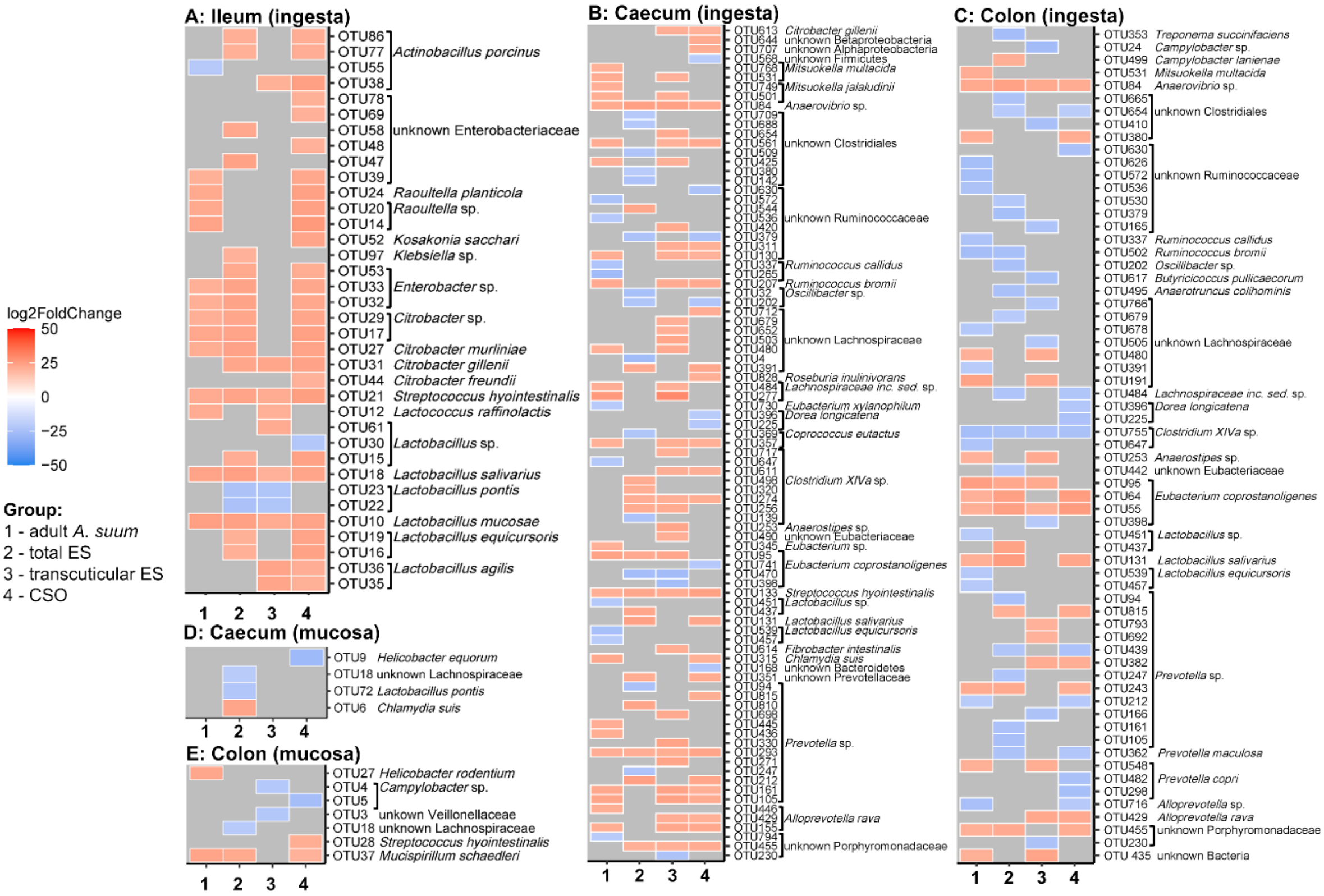

Supplementary Table S1. Results of group-wise PERMANOVA analyses on the microbiota composition of porcine ingesta and mucosa samples from the large intestine of pigs exposed to adult *Ascaris suum* worms or *A. suum* total excretory-secretory (ES), transcuticular ES, or cuticular somatic (CSO) antigens *vs* the control group. Significant *P*-values are printed in bold.

	Ingesta						Mucosa					
	**df**	**SS**	**MS**	**F**	**R^2**	***P***	**df**	**SS**	**MS**	**F**	**R^2**	***P***
**Adult *A. suum* *vs* control**												
Compartment (caecum *vs* colon)	1	0.07	0.07	1.20	0.06	**0.004**	1	0.15	0.15	2.94	0.13	**0.004**
Group	1	0.12	0.12	2.06	0.10	**0.004**	1	0.17	0.17	3.26	0.15	**0.004**
Residuals	17	1.01	0.06	-	0.84	-	16	0.83	0.05	-	0.72	-
Total	19	1.20	-	-	1.00	-	18	1.15	-	-	1.00	-
**Total ES *vs* control**												
Compartment (caecum *vs* colon)	1	0.09	0.09	1.29	0.07	**0.008**	1	0.14	0.14	1.50	0.09	0.141
Group	1	0.18	0.18	2.54	0.13	**0.023**	1	0.27	0.27	2.96	0.18	0.234
Residuals	16	1.12	0.07	-	0.81	-	12	1.08	0.09	-	0.73	-
Total	18	1.38	-	-	1.00	-	14	1.48	-	-	1.00	-
**Transcuticular ES *vs* control**												
Compartment (caecum *vs* colon)	1	0.09	0.09	1.03	0.06	**0.039**	1	0.20	0.20	3.40	0.15	**0.004**
Group	1	0.12	0.12	1.43	0.08	0.086	1	0.19	0.19	3.26	0.14	**0.004**
Residuals	15	1.28	0.09	-	0.86	-	16	0.94	0.06	-	0.71	-
Total	17	1.49	-	-	1.00	-	18	1.34	-	-	1.00	-
**CSO *vs* control**												
Compartment (caecum *vs* colon)	1	0.07	0.07	1.27	0.07	0.070	1	0.22	0.22	4.01	0.19	**0.016**
Group	1	0.10	0.10	1.77	0.10	0.344	1	0.17	0.17	3.10	0.15	**0.016**
Residuals	15	0.86	0.06		0.83	-	14	0.77	0.05	-	0.66	-
Total	17	1.03	-		1.00	-	16	1.16	-	-	1.00	-

Abbreviations: df, degrees of freedom; SS, sum of squares; MS, mean squares.

Supplementary Table S2. Summary of all model parameters of PCA or OPLS-DA models for metabolome data of pigs exposed to adult *Ascaris suum* worms or total *A. suum* excretory-secretory (ES), transcuticular ES, or cuticular somatic (CSO) antigens and an untreated control group.

Sample type/ compartment	Samples[1]	Model[2]	Components [3]	R2X [%][4]	Q2 [%][5]	R2Y [%][6]	CV-ANOVA[7]	Figure
**Ingesta**	all	PCA	18	98.3	95.5			6A,B
	all	OPLS-DA	3+0+0	84.2	8.27	3.85	0.7036	
Ingesta/jejunum	all	PCA	18	98.9	94.9			7A
	all	OPLS-DA	1+0+0	60.8	1.21	4.19	1	
Ingesta/ileum	all	PCA	8	96.9	89.2			7B
	all	OPLS-DA	1+0+0	53.1	-0.90	5.91	1	
Ingesta/caecum	all	PCA	2	62.2	49.3			7C
	all	OPLS-DA	1+0+0	38.0	3.66	9.65	1	
Ingesta/colon	all	PCA	7	90.6	68.8			7D
	all	OPLS-DA	-					
	Control *vs* adult *A. suum*	PCA	3	76.6	44.8			7E
	Control *vs* adult *A. suum*	OPLS-DA	2+1+0	69.8	23.2	84.8	0.7090	7F
**Serum**	all	PCA	5	84.4	55.3			7G
	all	(O)PLS-DA	-					

[1]The data analysis was conducted for all samples (all) or pairwise (e.g. control *vs* adult *A. suum*).
[2]PCA: principal component analysis; OPLS-DA: orthogonal partial least squares projection to latent structures-discriminant analysis.
[3]Components that capture variation found in X and Y are donated predictive. OPLS-DA: variation only found in X or Y are denoted orthogonal in X(OPLS) or Y(OPLS), respectively (e.g. 1+1+0: one predictive and one orthogonal in X and zero orthogonal in Y).
[4]Cumulative fraction of X variation modeled in that component, using the X model.
[5]Cumulative fraction of Y variation predicted by the X model in that component, according to cross-validation.
[6]Cumulative fraction of the Y variation modeled in that component, using the Y model.
[7]CV-ANOVA, ANalysis Of VAriance testing of Cross-Validated predictive residuals, is an analytical tool for judging the reliability of OPLS models.
